# The correlation between the coaptation height of mitral valve and mitral regurgitation after mitral valve repair

**DOI:** 10.1186/s13019-017-0687-0

**Published:** 2017-12-28

**Authors:** Dan Wei, Jie Han, Haibo Zhang, Yan Li, Chunlei Xu, Xu Meng

**Affiliations:** Department of cardiac surgery, Capital medical university affiliated Beijing anzhen hospital, Chaoyang District Anzhen Road No. 2, Beijing, 100029 China

**Keywords:** Coaptation Height of mitral valve, Mitral regurgitation, Mitral valve repair

## Abstract

**Background:**

To investigate the association between the coaptation height of mitral valve and mitral regurgitation after mitral valve repair.

**Methods:**

From Sep 2014 to Jun 2015, 20 patients underwent mitral valve valvuloplasty for mitral regurgitation were included. Ring annuloplasty was performed in all cases. Mitral valve short-axis dimension (MVd), coaptation height (CH), Left ventricular ejection fraction (LVEF) were measured by the transesophageal echocardiography before the operation in operation room and 3 months and 12 months after the operation by the transthoracic echocardiography. A degree from 0 to 4 was used to measure the degree of mitral regurgitation.

**Results:**

There were 14 patients with 0, 3 patients with 1, 3 patients with 2 of mitral regurgitation 12 months after the operation. CH (3.53 ± 1.91 mm) increased significantly at 3 months (5.05 ± 1.09 mm) and 12 months after operation (5.22 ± 1.15 mm) (*p* < 0.05). MVd and LVEF were not significantly changed after mitral valve repair. Furthermore, CH after resuscitation have a statistically significant negative correlation with the degree of mitral regurgitation 12 months after operation.

**Conclusion:**

The mitral valve repair with mitral valve ring induce the morphologic change of the mitral valve structure. The increase of CH after mitral valve repair may be one of the main factors in regulation of mitral regurgitation.

## Background

Mitral valve repair is a surgical procedure that has continuously developed through decades. Since 1968, Carpentier has developed the concept of prosthetic ring annuloplasty which aimed to restore the shape of the deformed annulus fibrosus of the MV [1]. With the development of the comprehension and evaluation of the pathology, standardization of the surgical techniques, the long term results from this surgery have gained a great progress. The remodeling of mitral annular provide the predictability and stability which has made MVP the most attractive approach on the treatment of mitral regurgitation in the United States [2, 3]. Nowadays, mitral valve repair (MVR) is the gold standard for severe degenerative mitral regurgitation (MR). Resection of the prolapsed leaflet has been widely performed and has shown excellent outcomes for posterior leaflet prolapse. The concept of leaflet preservation has become increasingly appreciated. In mitral valve repair, the greater the mitral valve coaptation area is, the lighter the mitral regurgitation is. Falk et al. found that chordal placement with minimal or no leaflet resection may contribute to better durability of MVR compared with leaflet resection, because of a longer zone of coaptation [4]. Post-repair coaptation length (CL) has been shown to be related to durability of MVR in patients with ischemic MR. [5] However, the association between post-repair CL and durability of MVR in degenerative MR has not been investigated. However, the exact value of the mitral valve coaptation area is not easy to obtain.

Doppler echocardiography was used to detect the functional information of transvalvular flow velocity, which could be used to measure the pressure gradient across valve and regurgitant flow [6]. Mitral valve area (MVA), a important functional index, can be detected by either 2-D echocardiography or Doppler pressure half-time [7]. In additional, transesophageal echocardiography (TEE) was also used to evaluate situation of the patients who has underwent percutaneous mitral BV in whom left atrial thrombus (LAT) is suspected [8] and for the intraoperative monitoring of the valvuloplasty procedure [9]. More recently, three-dimensional (3D) transoesophageal (TEE) echocardiography has provided accurate measurement of mitral valve morphology [10].

Therefore, in this study we used above methods to investigated whether it is possible to measure the degree of mitral regurgitation by measuring the mitral coaptation height. Accordingly, the present study aimed to evaluate the correlation between the height of mitral valve coaptation and mitral regurgitation after mitral valve repair.

## Methods

This was a retrospective study with the prospective follow-up of mitral valve regurgitation patients who underwent mitral regurgitation using a annuloplasty strip for mitral valve repair. This study was approved by Capital medical university affiliated Beijing anzhen hospital.

### Patients

A total of 20 patients were treated with the MV at Capital medical university affiliated Beijing anzhen hospital between Sep 2014 and Jun 2015. Transesophageal echocardiography (TEE) assessment assessment were routinely performed before the intervention to assess LVEF, mitral valve morphology and mitral regurgitation grade, and evaluation of factors that may contraindicate the procedure. Transthoracic echocardiographic (TTE) examination was performed after the operation. This study was approved by the ethics committee of Capital medical university affiliated Beijing Anzhen hospital. The informed consent was obtained form all the included patients.

### Operative procedure

All operations included in this study were performed via a median sternotomy approach. Cardiopulmonary bypass was established by ascending aortic and bicaval cannulation. The aorta was cross-clamped and cardiac asystole was achieved using intermittent antegrade and retrograde cardioplegia. Posterior leaflet prolapse was primarily repaired by leaflet resection. The resection size and shape were determined by a surgeon in charge of each case. Neochordal placement was added if necessary. Ring annuloplasty was performed in all cases. The type of ring was chosen based on surgeon’s preference. The size of ring was determined by sizing the area of anterior leaflet using prosthetic sizers made by each manufacturer.

The surgical procedures with prosthetic ring annuloplasty were performed in all patients. Prosthetic ring annuloplasty was performed in 5 cases with 30-mm prosthetic ring (Edwards physio 2), 8 cases with 32-mm prosthetic ring (Edwards 5200), 5 cases with 34-mm prosthetic ring (Edwards physio 2) and 2 cases with 36-mm prosthetic ring (Edwards physio 4450).

### Echocardiographic measurements

**Fig. 1:**
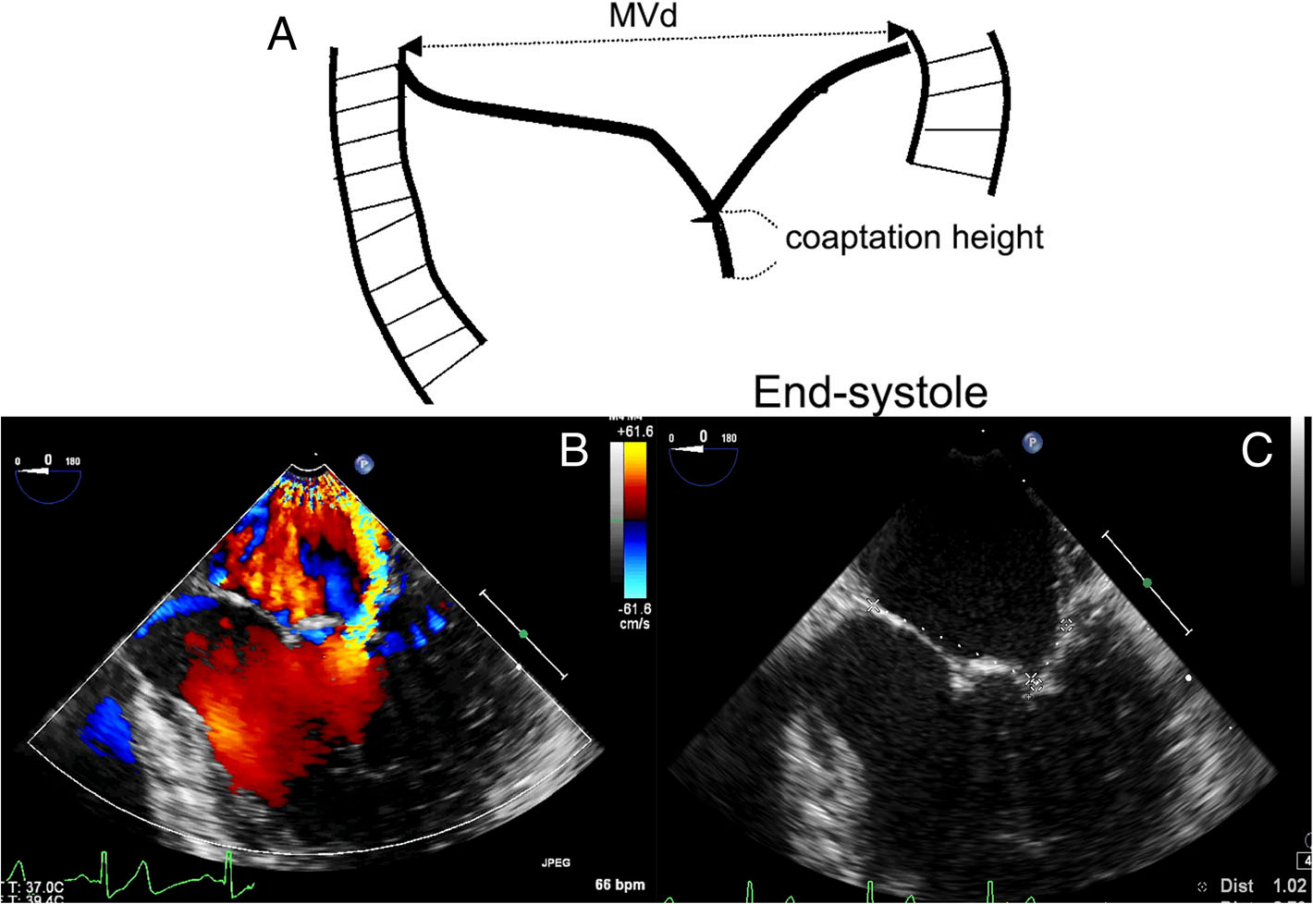
The TEE view for measurement parameter. **a** Coaptation height measurement. **b** Measurement under TEE. **c** Coaptation height measurement by TEE

TTE was performed using commercially available ultrasound systems (E33; Philips, Netherlands) equipped with an S5–1 transducer, and 2D, M-mode and Doppler data were acquired with the patient in the left lateral decubitus position. Transthoracic echocardiography was performed at 3 months and 12 months postoperatively. TEE with X7-2 t transducer was applied. The TEE view for the measurement was 3-chamber view and the middle square of mitral apparatus in short axis (Fig. 1). Mitral valve short-axis dimension (MVd) and Coaptation height (CH) at endsystole were measured (Fig. 1). The coaptation height was defined as the length between the free edge of the leaflet and the anterior and posterior lobes to left atrial surface level at end-systole stage. Carpentier typing was used to unify the mitral leaflet partition. The posterior lobe is divided into three leaves, the lateral fan as P1, the middle fan leaf as P2 and the medial fan leaf as P3. The corresponding anterior lobe was also divided into three parts, the lateral 1/3 part as A1, the middle 1/3 as A2, the medial 1/3 as A3. 20 cases in the operation room under anesthesia were examined by TEE.The Height of the corresponding section were set. The A2P2 was measured by four chamber view of the middle esophagus. The A1P1 was measured by five chamber view of the middle esophagus. The A3P3 was measured by deep esophageal short four chamber view showing coronary sinus. Three cardiac cycles were selected to measure the corresponding height. The mitral regurgitation grade was determined according to the following scale: 0, no or trivial mitral regurgitation; 1+, mild; 2+, mild to moderate mitral regurgitation; 3+, moderate to severe mitral regurgitation; and 4+, severe mitral regurgitation. The coaptation height (the longest coaptation height of the anterior and posterior leaflets) was measured in early systole.

### Statistical analysis

The values obtained were analyzed with Student’s T test. Linear regression analysis was determined using the least squares method. A *p* value <0.05 was considered as indicative of significant significance. Data are presented as the means ± standard deviation (SD).

## Results

### Baseline characteristics

The mean age of all patients was 52.45 ± 14.26 years and 11 patients (55%) were women. In the 20 cases studied, mitral regurgitation was degree 1 in 2 patients, degree 2 in 4 cases, and degree 3 in 14 cases before the operation. All patients had a posterior middle scallop prolapse, the mean LVEF was 62.75 ± 7.94%. All patients underwent MVP with prosthetic ring. Pre-operative and operative data are shown in Table 1. In the 20 cases studied, mitral regurgitation was 0 in 14 patients, 1 in 3 cases, and 2 in 3 cases 12 months after the operation.

**Table 1:** Measurement values

		MR (*n* = 20)
Age (years)		52.45 ± 14.26
Weight (kg)		67.8 ± 8.46
Height (cm)		164.7±8.20
Perfusion time (min)		40.4 ± 12.1
Cross-clamp time (min)		31.7 ± 14.2
LVEF (%)	Before the surgery	62.75 ± 7.94
	3 months after surgery	61.05 ± 9.00
	12 months after surgery	62.8 ± 10.52
MVd (mm)	Before the surgery After heart resuscitation	30.83 ± 3.48 31.95 ± 4.56
	3 months after surgery	33.33 ± 3.49
	12 months after surgery	33.78 ± 3.87

Left ventricular ejection fraction (LVEF), mitral valve short-axis dimension (MVd)

Values were shown as mean ± standard deviation

### Leaflet morphologic change and Echocardiographic factor in regulating mitral regurgitation

**Fig. 2:**
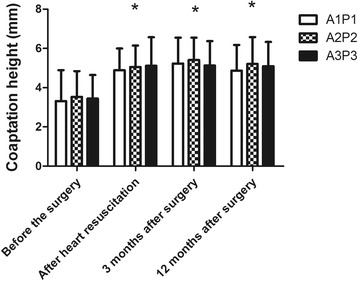
The preoperative and postoperative value of coaptation height

**Fig. 3:**
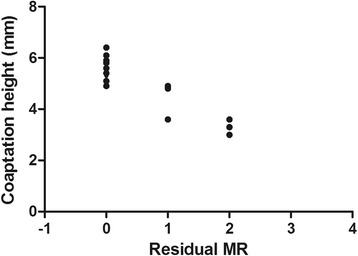
The relationship between the coaptation height and residual regurgitaion

Table 1 shows the preoperative and postoperative values of EF, MVd. The values of LVEF and MVd were not significantly changed. However, MVd showed only increase trend after the surgery. In Fig. 2, CH of A1P1, A2P2, A3P3 was increased significantly. Among those stated index, the average values of CH (A1P1, A2P2, A3P3) after heart resuscitation showed a statistically significant negative correlation with degree of mitral regurgitation 12 months after the operation. (*p* < 0.05 *r* = 0.81) (Fig. 3).

## Discussion

Surgical valve repair for mitral regurgitation (MR) has significant advantages over valve replacement and has acquired greater importance as a surgical treatment of MR. Some researches have already confirmed that the mitral valve repair could provided great long term results for the patients with mitral insufficiency [11, 12]. Although favorable clinical and functional results have been reported, the exact information of the valve leaflet morphology after those repairs is limited. The coaptation height was considered as an important morphologic index for mitral regurgitation. However, there are no uniform standards for the value of the coaptation height, which was mainly caused by the difficulty in measurement of the exact coaptation height in clinical practice, while even the exact point of the edge of free zone of leaflet at coaptation was difficult to obtained.

The main results of the our study showed that the LVEF and MVd were not significantly influenced after mitral valve repair. However, the coaptation height was significantly increased after the operation. Interestingly, CH after heart resuscitation showed a statistically significant negative correlation with degree of mitral regurgitation 12 months after operation, which meant CH after heart resuscitation may be an important predictive factor for the post-operative mitral regurgitation. Some other researches have also investigated the change of the mitral valve morphology after mitral annuloplasty. It was reported that the mitral annuloplasty with ring could significantly improve the stresses and the valve coaptation with annular dilatation [13]. The reconstruction of the posterior leaflet compressive stresses and nearnormal coaptation was crucial to the mitral annuloplasty. It was also reported that the anuloplasty ring could significantly improve the mitral valve coaptation by reducing the delayed action of the leaflet and preventing the mitral regurgitation in the case of acute left ventricular ischemia after ring implantation [14]. In a total, the normal coaptation is very important in the mitral annuloplasty.

There were also some limitations in our study. The number of the cases in our study is small. Further study with lager numbers is still needed. The differences of the surgery procedures and cardiac function were not excluded in this study, which may affect the measurement of mitral regurgitation.

## Conclusion

In conclusion, mitral valve repair could induce the morphologic change of the mitral valve structure. The increase of CH after mitral valve repair may be one of the main factors in regulation of regurgitation after mitral valve repair.
